# Imaging in pediatric appendicitis is key to a low normal appendix percentage: a national audit on the outcome of appendectomy for appendicitis in children

**DOI:** 10.1007/s00383-018-4244-2

**Published:** 2018-03-09

**Authors:** M. D. Bolmers, C. C. van Rossem, R. R. Gorter, W. A. Bemelman, A. A. W. van Geloven, H. A. Heij, On behalf of the Snapshot Appendicitis Collaborative Study group

**Affiliations:** 1 Department of Surgery, Tergooi Hospital Hilversum, P.O. Box 10016, 1201 DA Hilversum, The Netherlands; 2 Department of Surgery, Maasstad Hospital, Rotterdam, The Netherlands; 3 Department of Pediatric Surgery, Academic Medical Center, VU Medical Center, Amsterdam, The Netherlands; 4 Department of Surgery, Academic Medical Center, Amsterdam, The Netherlands

**Keywords:** Appendicitis, Children, Appendectomy, Pediatric

## Abstract

**Purpose:**

A laparoscopic approach for emergency appendectomy is increasingly used, in pediatric patients as well. The objective of this study is to audit the current state of diagnostic work-up, surgical techniques and its outcome in children with acute appendicitis.

**Methods:**

A prospective consecutive observational cohort study was carried out in a 2-month study period. All patients under 18 years that were operated for suspected acute appendicitis were included. Primary outcome was the infectious complication rate after open and laparoscopic approach; secondary outcomes were preoperative use of imaging and post-operative predictive value of imaging, normal appendix rate and children with a postoperative ileus.

**Results:**

A total of 541 children were operated for suspected acute appendicitis in 62 Dutch hospitals. Preoperative imaging was used in 98.9% of children. The normal appendix rate was 3.1%. In 523 children an appendectomy was performed. Laparoscopy was used in 61% of the patients and conversion rate was 1.7%. Complicated appendicitis was diagnosed in 29.4% of children. Overall 30-day complication rate was 11.9% and similar after open and laparoscopic. No difference was found in superficial surgical site infections, nor in intra-abdominal abscesses between the open and laparoscopic approach. Complicated appendicitis is an independent risk factor for infectious complications.

**Conclusion:**

The laparoscopic approach is most frequently used, except for young children. Superficial surgical site infections are more frequent after open surgery only in patients with complicated appendicitis. The normal appendix rate is low, most likely because of routine preoperative imaging.

## Introduction

In children, emergency appendectomy is the most performed acute surgical procedure [1].

Two options are available to perform an appendectomy: the open approach through a gridiron incision first described by McBurney in 1894 [2], and the laparoscopic approach described by de Kok [3] in 1977.

Almost 25% of all appendectomies are performed in children and appendicitis has a lifetime incidence of 7–8% with a peak in adolescents [4]. Diagnostic workup and treatment can be different in children and adults.

Over recent years overall diagnostic work-up and treatment are changing. There is an increased use of preoperative imaging to reduce the negative appendectomy rate and new imaging techniques are implemented. Regarding treatment, laparoscopy is more frequently used, but is still not universally accepted as standard of care in the treatment of appendicitis in children. In children, a recent meta-analysis showed a broad equivalence in uncomplicated appendicitis and an increased risk of intra-abdominal abscess in laparoscopy in case of complicated appendicitis [5].

Recently the idea is endorsed that, instead of being a progressive disease starting with uncomplicated stage and evolving to a complicated form, complicated and uncomplicated appendicitis are now considered as two different entities [6]. This has led to new studies evaluating the conservative treatment with antibiotics of uncomplicated appendicitis in children as well as adults [7, 8].

As a part of a nationwide cohort study on surgical treatment of acute appendicitis in The Netherlands [9] this study’s objectives were to evaluate the national guideline and to assess the variation in practice and outcome in the treatment of acute appendicitis in children. Emphasis will be on infectious complications and its clinical consequences after open and laparoscopic appendectomy.

## Methods

### Study design

A consecutive observational cohort study (snapshot) was performed in hospitals in the Netherlands. All hospitals that provided acute surgical care were invited to participate. This included academic, pediatric and general community hospitals (teaching and non-teaching). The study was designed and led by surgical residents, who together with house officers collected the data.

All consecutive patients undergoing surgery for suspected acute appendicitis were included between June and July 2014. This period was preceded by a pilot phase in May in 8 hospitals in the Amsterdam region. Only pediatric patients (age below 18 years) were analyzed in this study. Patients were excluded if they underwent an elective appendectomy (either interval or as a routine procedure, for instance, in patients with malrotation). Furthermore, patients initially treated non-operatively were also excluded, even though some of them underwent a subsequent appendectomy. All patients that underwent diagnostic laparoscopy without removal of the appendix, or patients that had a different procedure, but were initially operated for suspected appendicitis were included to evaluate the normal appendix rate. A normal appendix was diagnosed by the operating surgeon when there was no sign of inflammation (rigid appendix, prominent vascularization, free fluid, perforation).

Patients were treated according to the local protocol of the participating hospitals, based on the national guideline. No adjustments on treatment were imposed for participating hospitals. No additional follow-up or contribution was asked for the patients.

Primary outcome was complication rate, mainly infectious complications [superficial surgical site infection (SSI) and intra-abdominal abscess (IA)] after open and laparoscopic appendectomy. Secondary outcomes were preoperative use of imaging and post-operative predictive value of imaging, normal appendix rate and children with a postoperative ileus. SSI was defined as the clinical suspicion for a SSI that required additional interventions (either antibiotics or drainage). IA was defined as an intra-abdominal fluid collection that required antibiotics or drainage procedures (surgical or percutaneous). Ileus was defined as no resumption of diet within 5 days [10]. Preoperative definition of complicated appendicitis consisted of signs of perforation in the radiology report, i.e., free fluid, free air or abscess. Intra-operative definition of complicated appendicitis consisted of the presence of perforation or necrosis of the appendix.

### Data collection

Data were collected from presentation at the emergency room until 30 days after surgery.

Data were anonymized and entered into a web-based database by a single local investigator. Data were extracted from the electronic patient database system, admission charts and operative reports or directly from the operating surgeon when details were unclear. To identify complications during follow-up, the electronic patient database system was monitored to detect postoperative attendance to the emergency department, unscheduled outpatient clinic visits, hospital readmissions, imaging or intervention. Additional checking of admission diagnosis and surgical procedures in the study months identified any possible missing patients.

### Statistics

Statistical analysis was performed using SPSS^®^ version 22 (IBM, Armonk, New York, USA).

All normally distributed variables were analyzed using Student’s *t* test. *χ*^2^ test or Fisher’s exact test where appropriate were used in dichotomous outcomes. Univariable odds ratios were calculated to compare complications between laparoscopic and open appendectomy.

Analysis was performed according to the intention to treat principle and converted patients were therefore analyzed in the laparoscopic group.

## Results

### Included patients

A total of 541 patients were included from 62 Dutch hospitals, including all 7 pediatric hospitals.

**Fig. 1:**
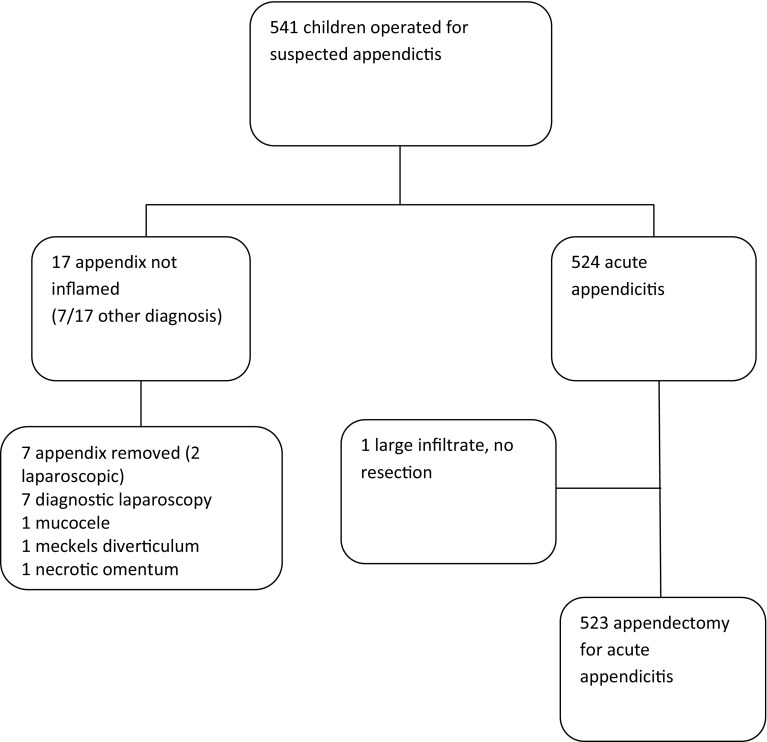
Flow diagram

Number of children ranged from 1 to 27 per hospital. In 17 patients the appendix was peroperatively not inflamed (3.1%), an alternative diagnosis was noted in 7 of them (41.2%), and all of them had preoperative imaging. The appendix from only 7 of the 17 patients was removed mainly because of the open approach. Unexpectedly, in 3 of these patients the pathology report stated uncomplicated appendicitis. One of the patients with an acute appendicitis was treated without appendectomy, leaving a total of 523 patients that underwent an appendectomy for suspected appendicitis (Fig. 1). The outcomes of these procedures were examined.

### Preoperative data and imaging

Demographic and imaging characteristics are displayed in Table 1. The majority of the children were boys (60%) and the median age was 12 years. As expected, appendicitis under the age of 6 was rare; only 28 patients (5.4%). The majority was treated in a general community hospital, teaching or non-teaching (91.6%). 8.4% in a pediatric (academic) hospital, of which 16.1% children under the age of 6.

**Table 1:** Demographic and imaging

	Total (*N* = 523)	Laparoscopy (ITT) (*N* = 319)	Open (*N* = 204)	P*
Age				< 0.001
0–5	28 (5.4)	10 (3.1)	18 (8.8)	
6–11	212 (40.5)	105 (32.9)	107 (52.5)	
12–17	283 (54.1)	204 (63.9)	79 (38.7)	
Gender				0.005
Male	314	177 (55.5)	137 (67.2)	
Female	209	142 (44.5)	67 (32.8)	
Day since onset complaints				0.347
Median	1	1	1	
<3 days		240 (77.4)	161 (79.3)	
≥ 3 days		70 (22.6)	42 (20.7)	
Missing	10			
Institution				
Academical/childrens	44 (8.4)	32 (10.0)	12 (5.9)	
Community	479 (91.6)	287 (90.0)	192 (94.1)	
Imaging				0.399
Ultrasound only	481 (92)	288 (90.3)	193 (94.6)	
Ultrasound + CT-scan	7 (1.3)	6 (1.9)	1 (0.5)	
Ultrasound + MRI scan	28 (5.4)	20 (6.3)	8 (3.9)	
MRI only	1 (0.2)	1 (0.3)	0 (0)	
No imaging	6 (1.1)	4 (1.3)	2 (1.0)	
Imaging conclusion				0.012
Uncomplicated	293 (76)	238 (75.6)	155 (76.7)	
Complicated	77 (14.9)	40 (12.7)	37 (18.3)	
Not conclusive	47 (9.1)	37 (11.7)	10 (5.0)	

*χ2 test or Fisher’s exact test where appropriate

In nearly all patients preoperative imaging was performed, (98.9%). Ultrasound was used as the only imaging modality in 92%. Complementary CT-scan or MRI scan was obtained in 1.3 and 5.4%, respectively. Complicated appendicitis was suspected preoperatively in 14.9% of the patients. An inconclusive result from preoperative imaging was in 9.1% of patients. Complex (or complicated) appendicitis on imaging was defined as signs of perforation described in the radiology report, e.g., free fluid or abscess formation.

### Intraoperative data

Intraoperative data are displayed in Table 2. A laparoscopic appendectomy was performed in 319 children (61%), although in 1.7% of these procedures it was necessary to convert to open appendectomy. The distribution of the application of laparoscopy for each age group is as follows: 35.7% of children under 6, between 6 and 11 years in 49.5 and 72.1% of children between 12 and 18 years. Conversion rates were 0, 1.4 and 2.1%, respectively.

**Table 2:** Operative data

	Total (*N* = 523)	Laparoscopic (*N* = 319)	Open (*N* = 204)	P*
Conversion		9 (1.7)		
Surgeon				0.380
Consultant or resident under supervision	452 (86.4)	274 (85.9)	178 (87.3)	
Resident alone	71 (13.6)	45 (14.1)	26 (12.7)	
Operating time (mean, min)	39.34	42.90	33.40	0.001^#^
Hospital stay (median, days)	2	2	2	0.473^#^
(Range)	(1–30)	(1–16)	(1–30)	
Intraoperative diagnosis				0.457
Simple	369 (70.6)	224 (70.2)	145 (71.1)	
Complicated	154 (29.4)	95 (29.8)	59 (28.9)	
Antibiotics				0.603
No prophylaxis	12 (2.3)	9 (2.8)	3 (1.5)	
Intraoperative	374 (71.5)	227(71.2)	147 (72.1)	
Continued	(26.2)	83 (26.0)	54 (26.5)	
Histology				0.302
Normal	14 (2.7)	12 (3.8)	2 (1.0)	
Simple	404 (77.2)	244 (76.5)	160 (78.4)	
Necrosis	42 (8.0)	26 (8.2)	16 (7.8)	
Perforation	43 (8.2)	23 (7.2)	20 (9.8)	
Neoplasm	3 (0.6)	2 (0.6)	1 (0.5)	
Other	9 (1.7)	5 (1.6)	4 (2.0)	
No pathology	8 (1.5)	7 (2.2)	1 (0.5)	

*χ^2^ test or Fisher’s exact test where appropriate

^#^Mann–Whitney *U* test

A laparoscopic operation was performed in 56.4% of the boys and in 67.9% of the girls.

In patients with preoperative suspicion of uncomplicated appendicitis on imaging, 83.7% were diagnosed with uncomplicated appendicitis during operation. In patients with preoperative suspicion of complicated appendicitis on imaging, 71.4% were diagnosed with complicated appendicitis during operation.

Intraoperatively, in 29.4% of the patients, complicated appendicitis was diagnosed. In 5.2% of patients with complicated appendicitis undergoing a laparoscopic appendectomy conversion was necessary.

Complicated appendicitis was diagnosed significantly more frequent in children < 6-years-old compared to children 6–11 years old and between 12 and 18 years old; 67.9 vs 29.7 vs 25.4%, respectively (*P* < 0.001).

The overall complication rate after an appendectomy for acute appendicitis was 11.9% during the 30-day follow-up. The rates between laparoscopic and open approaches were comparable (OR 1.064; 95% CI 0.620–1.828; *P* = 0.462). In complicated appendicitis overall complication rate was 28.6% (OR 7.800; 95% CI 4.329–14.053; *P* = 0.001). Pediatric surgeons had similar laparoscopic approach rates (OR 1.584; 95% CI 0.645–3.890) and complication rates (OR 0.644; 95% CI 0.213–1.949) as other surgeons.

### Infectious complications

After an appendectomy the overall rate of SSI was 1.7%, of IA was 5.9% and of an ileus was 2.5%.

In Table 3, the overall complication rates for laparoscopic and open appendectomy are displayed. No statistical difference could be detected for SSI, 0.9 vs 2.9% (OR 3.192; 95% CI 0.790–12.91; *P* = 0.087). There was also no significant difference in IA, 6.0 vs 5.9%, respectively (OR 0.987; 95% CI 0.468–2.079; *P* = 0.560), nor could it be detected in ileus, 2.2 vs 2.9% (OR 1.351; 95% CI 0.447–4.077; *P* = 0.395).

**Table 3:** Overall 30 day complications

	Total	Laparoscopic	Open	Odds ratio (95% CI)	P*
Any complication	62 (11.9)	37 (11.6)	25 (12.3)	1.064 (0.620–1.828)	0.462
Infectious complication					
Any infectious	39 (7.5)	22 (6.9)	17 (8.3)	2.364 (0.633–2.364)	0.331
Superficial surgical site infection	9 (1.7)	3 (0.9)	6 (2.9)	3.192 (0.790–12.91)	0.087
Intra-abdominal abscess	31 (5.9)	19 (6.0)	12 (5.9)	0.987 (0.468–2.079)	0.560
Ileus	13 (2.5)	7 (2.2)	6 (2.9)	1.351 (0.447–4.077)	0.395
Hospital stay (days) (median)	541	2 (1–17)	2 (1–31)		0.835^#^
Hospital re-admittance	30 (5.7)	20 (6.3)	10 (4.9)	1.682 (0.353–1.682)	0.326
Re-operation	13 (2.5)	10 (3.1)	3 (1.5)	0.461 (0.125–1.696)	0.184

*χ^2^ test or Fisher’s exact test where appropriate

^#^Mann–Whitney *U* test

We have displayed the outcome in specific disease severity group in Table 4. In complicated appendicitis the overall rate of SSI was 2.6%, of IA was 18.2% and of an ileus was 8.4% of the patients. We noticed that the SSI rate in the laparoscopic appendectomy group was significantly lower compared to the open appendectomy group; 0 vs 6.8% (OR 1.030; 95% CI 0.170–6.242; *P* = 0.020). No statistical difference was found for IA; 17.9 vs 18.6% (OR 1.051; 95% CI 0.454–4.434; *P* = 0.535), nor for ileus, 7.4 vs 10.2% (OR 1.423; 95% CI 0.454–4.461; *P* = 0.372).

**Table 4:** 30-day complications in complicated appendicitis

	Total	Laparoscopic	Open	Odds ratio (95% CI)	P*
Any complication	44 (28.6)	25 (26.3)	19 (32.2)	1.330 (0.653–2.710)	0.466
Infectious complication					
Any infectious	32 (20.8)	17 (17.9)	15 (24.4)	1.564 (0.712–3.435)	0.180
Superficial surgical site infection	4 (2.6)	0 (0.0)	4 (6.8)	1.030 (0.170–6.242)	0.020
Intra-abdominal abscess	28 (18.2)	17 (17.9)	11 (18.6)	1.051 (0.454–2.434)	0.535
Ileus	13 (8.4)	7 (7.4)	6 (10.2)	1.423 (0.454–4.461)	0.372
Hospital re-admittance	21 (13.6)	14 (14.7)	7 (11.9)	0.779 (0.295–2.058)	0.401
Re-operation	10 (6.5)	8 (8.4)	2 (3.4)	0.382 (0.078–1.862)	0.187

*χ^2^ test or Fisher’s exact test where appropriate

Factors of increasing the risk of any infectious complication are listed in Table 5. Children under 6 had a higher chance of developing an IA (*P* = 0.002). No difference in infectious complications was found in the other age groups. There was no difference in open or laparoscopic approach with regard to developing an IA (*P* = 1.000).

**Table 5:** Infectious complication causes

	Superficial surgical site infection	P*	Intra-abdominal abscess	P*
Approach		0.087		0.566
Laparoscopic	3 (0.9)		19 (6.0)	
Open	6 (2.9)		12 (5.9)	
Age		0.448		0.002
0–5	1 (3.6)		6 (21.4)	
6–11	5 (2.4)		12 (5.7)	
12–17	3 (1.1)		13 (4.6)	
Gender				0.334
Male	6 (1.9)		17 (5.4)	
Female	3 (1.4)		14 (6.7)	
Day since onset complaints median		0.352		0.001
<3 days	6 (1.5)		13 (3.2)	
≥ 3 days	3 (2.5)		18 (14.8)	
Migration of pain		0.753		0.223
Yes	3 (1.4)		8 (3.8)	
No	6 (2.0)		22 (7.5)	
Temperature		0.632		0.100
≤37.4	3 (1.2)		7 (2.9)	
>37.4	5 (2.0)		23 (9.2)	
Leucocytes (mmol/ml)		0.219		0.142
≤ 13.6	1 (0.5)		7 (3.5)	
> 13.6	8 (2.5)		24 (7.5)	
CRP		0.235		0.001
≤ 39	3 (1.0)		5 (1.6)	
> 39	6 (2.9)		26 (12.6)	
Imaging conclusion		0.035		0.001
Uncomplicated	5 (1.3)		13 (3.3)	
Complicated	4 (5.2)		13 (16.9)	
Not conclusive	0 (0)		4 (8.5)	
Intra-operative diagnosis		0.256		0.001
Complicated	4 (2.6)		28 (18.2)	
Simple	5 (1.4)		4 (0.8)	
Surgeon		0.266		0.371
Consultant or resident with supervision	9 (2.0)		28 (6.2)	
Resident alone	0 (0.0)		3 (4.2)	
Operating time		0.714		0.002
< 60 min	8 (1.9)		18 (4.2)	
≥ 60 min	1 (1.6)		9 (14.8)	
Missing	0		4 (11.4)	
Antibiotics		0.427		0.001
No	0 (0.0)		0 (0.0)	
Perioperative < 24 h	5 (1.3)		3 (0.8)	
Prolonged for complicated appendicitis	4 (2.9)		28 (20.4)	

*χ^2^ test or Fisher’s exact test where appropriate

In binary logistic regression analysis no difference was found in overall incidence of surgical site infections (OR 0.313; 95% CI 0.77–1.267; *P* = 0.104) or intra-abdominal abscesses (OR 1.013; 95% CI 0.481–2.135; *P* = 0.972) between the laparoscopic or open approach.

In multivariate analysis, complicated appendicitis shows to be the only independent risk factor for any infectious complication, Table 6.

**Table 6:** Multivariate analysis for infectious complications

	OR	95% CI	*P*
Approach	0.900	0.409–1.982	0.794
Age < 6	2.614	0.829–8.241	0.101
Complicated appendicitis	10.770	3.978–29.157	0.001
Imaging results	1.856	0.815–4.229	0.141

Multivariate binary logistic regression

## Discussion

This national cohort study shows that since the introduction of our national guideline, the majority of children undergo preoperative imaging studies. This has led to a low normal appendix rate of 3.1%. Furthermore, there was no significant overall difference in SSI and IA between the laparoscopic and open approach in children with acute appendicitis. However, we found a significant difference in infectious complications between complicated and uncomplicated appendicitis. Our study shows less SSI in the laparoscopic approach with no increase in the IA for patients with complicated appendicitis. Complicated appendicitis is identified as the sole risk factor for any infectious complication.

In literature, inconsistent results have been published both in the adult and pediatric population regarding the potential benefits and harms of laparoscopic appendectomy. A Cochrane review reported a slightly lower incidence of superficial surgical site infections in the laparoscopic technique, but an increased risk (nearly twofold) of IA after laparoscopic appendectomy, although results should be interpreted with care due to the quality of this studies [11]. In children, the same hazard of IA is noted after laparoscopic appendectomy after complicated appendicitis in several meta-analysis although large, well-designed RCTs on this subject in children are needed [5, 12]. Our results are comparable with other studies mentioning that the surgical approach does not affect the incidence of intra-abdominal abscesses, but the severity of the appendicitis (uncomplicated versus complicated) does [13].

We showed a low normal appendix rate. This is most likely due to the high level of preoperative imaging and accuracy. The Dutch appendicitis guideline was implemented in 2010. In our study, the first post guideline, almost all children had preoperative imaging and, in accordance with these national guidelines, ultrasound was most commonly used. Before implementation of the guidelines a baseline survey showed a normal appendix rate of 15.9%, it also showed that preoperative imaging was performed in only 44.2% [14]. Although appendicitis scoring systems like the Alvarado score can be of aid, they are known to be not accurate enough to predict acute appendicitis in children [15].

CT, and in children mainly MRI, are known for high sensitivity in diagnosing appendicitis [16, 17]. But costs, radiation exposure with potential negative effects from CT-scan and availability need to be considered. New combined diagnostic strategies are being developed to increase sensitivity as far as possible with respect to cost and availability [18], and recent studies show that mandatory imaging in suspected appendicitis is cost efficient [19, 20].

In 50% of children with a intraoperative normal appendix the preoperative imaging was inconclusive for appendicitis, therefore surgery, in particular laparoscopy, can be seen as part of the diagnostic algorithm.

Our study is in line with other studies that showed that decreasing age comes with an increase in complicated appendicitis, this could be because of a diagnostic challenge in very young children [21, 22], or because complicated appendicitis is a different entity and more frequently seen in young children. As a consequence the IA rate is also profoundly increased in this group.

Our study shows that although the majority of all children are operated laparoscopically, the open approach is mostly applied in children under 6-years-old. The difference in approach in the youngest age group might be because of use of specific pediatric instruments, or expected difficulty of procedures. Although the laparoscopic approach has therapeutic and diagnostic advantages in the general population [23, 24], in young children these advantages have so far never been proved although faster recovery is frequently reported in laparoscopy [25]. The high percentage of boys in our population (60%), was a surprise and cannot satisfactorily be explained.

Nowadays complicated appendicitis is believed to be a different entity than uncomplicated appendicitis [6]. In this cohort 28.7% of cases were defined as complicated appendicitis, and it was the only independent risk factor for infectious complications. Complicated appendicitis was equally divided between laparoscopic and open approach, as was the preoperative suspicion of complicated appendicitis on imaging and the final histological conclusion. In complicated appendicitis a higher rate of SSI was found in the open technique, but no difference in IA. In our opinion this is explained by the fact that during open appendectomy the layers of the abdominal wall are exposed to the inflamed appendix and in some cases also purulent fluid. In the laparoscopic approach the abdominal wall is protected. Other complications were equally distributed. Overall conversion rate was very low, 1.7%, and in complicated appendicitis a 5.2%. Conversion rate is much lower than historical cohorts reported [26, 27], this could be because in these historical cohorts surgeons may still have been in there learning curve. Conversion in our study was mostly because of a large abscess or infiltrate with insufficient overview.

More and more studies are being published on conservative treatment of acute appendicitis, comparing initial non-operative treatment with antibiotics versus appendectomy in uncomplicated appendicitis, which might be a safe option [7, 28, 29]. To start the appropriate treatment regime, correct preoperative discrimination between uncomplicated and complicated appendicitis with the help of imaging studies is crucial. In this prospective cohort study all consecutive children undergoing appendectomy for suspected acute appendicitis were included. This provides a veracious overview of the current treatment of appendicitis and the complications of appendectomy for acute appendicitis, whereas randomized trials use strict in and exclusion criteria. The downsides of this cohort study, a wide variety of treatment modalities were used in the participating centers, and the risk of inter observer variation due to the multiple operating surgeons were minimized by the appointment of one local researcher. This local researcher was responsible for data collection and entry.

Our study shows that laparoscopic approach in pediatric appendicitis is safe, with no overall differences in infectious complications compared to the open approach.

The routine use of preoperative imaging, as dictated by national guidelines, most likely results in a low normal appendix rate in children. Although imaging accuracy was acceptable, it was, in multivariable analysis, not able to predict complicated appendicitis. This needs to be improved to safely expand studies of non-operative treatment regimes.

Complicated appendicitis is identified as the only independent risk factor for infectious complications and the laparoscopic, when compared to the open approach in complicated appendicitis, causes less SSI and an equal incidence of IA.
